# 2′,3′,4′-Trihydroxychalcone changes estrogen receptor α regulation of genes and breast cancer cell proliferation by a reprogramming mechanism

**DOI:** 10.1186/s10020-022-00470-z

**Published:** 2022-04-25

**Authors:** Candice B. Herber, Chaoshen Yuan, Anthony Chang, Jen-Chywan Wang, Isaac Cohen, Dale C. Leitman

**Affiliations:** 1 Department of Nutritional Sciences and Toxicology, University of California, Berkeley, CA 94720-3104 USA; 2 Iaterion, University of California, QB3, 1700 4th Street Byers Hall, Suite 214, San Francisco, CA 94158 USA; 3 Present Address: DENALI Therapeutics, 161 Oyster Point Blvd, South San Francisco, CA 94080 USA; 4 Present Address: Biomedical Sciences Program, University of California San Francisco, San Francisco, CA 94143 USA

**Keywords:** Breast cancer, Estradiol, Estrogen, Estrogen receptor, Cell proliferation, Menopause hormone therapy, Selective estrogen receptor modulator, Chalcone, MCF-7 cells

## Abstract

**Background:**

Menopausal hormone therapy (MHT) is recommended for only five years to treat vasomotor symptoms and vulvovaginal atrophy because of safety concerns with long-term treatment. We investigated the ability of 2′,3′,4′-trihydroxychalcone (2′,3′,4′-THC) to modulate estrogen receptor (ER)-mediated responses in order to find drug candidates that could potentially prevent the adverse effects of long-term MHT treatment.

**Methods:**

Transfection assays, real time-polymerase chain reaction, and microarrays were used to evaluate the effects of 2′,3′,4′-THC on gene regulation. Radioligand binding studies were used to determine if 2′,3′,4′-THC binds to ERα. Cell proliferation was examined in MCF-7 breast cancer cells by using growth curves and flow cytometry. Western blots were used to determine if 2′,3′,4′-THC alters the E2 activation of the MAPK pathway and degradation of ERα. Chromatin immunoprecipitation was used to measure ERα binding to genes.

**Results:**

The 2′,3′,4′-THC/E2 combination produced a synergistic activation with ERα on reporter and endogenous genes in human U2OS osteosarcoma cells. Microarrays identified 824 genes that we termed reprogrammed genes because they were not regulated in U2OS-ERα cells unless they were treated with 2′,3′,4′-THC and E2 at the same time. 2′,3′,4′-THC blocked the proliferation of MCF-7 cells by preventing the E2-induced activation of MAPK and c-*MYC* transcription. The antiproliferative mechanism of 2′,3′,4′-THC differs from selective estrogen receptor modulators (SERMs) because 2′,3′,4′-THC did not bind to the E2 binding site in ERα like SERMs.

**Conclusion:**

Our study suggests that 2′,3′,4′-THC may represent a new class of ERα modulators that do not act as a direct agonists or antagonists. We consider 2′,3′,4′-THC to be a reprogramming compound, since it alters the activity of ERα on gene regulation and cell proliferation without competing with E2 for binding to ERα. The addition of a reprogramming drug to estrogens in MHT may offer a new strategy to overcome the adverse proliferative effects of estrogen in MHT by reprogramming ERα as opposed to an antagonist mechanism that involves blocking the binding of estrogen to ERα.

**Supplementary Information:**

The online version contains supplementary material available at 10.1186/s10020-022-00470-z.

## Introduction

In women of reproductive age, estrogen is mainly synthesized in the granulosa cells of ovarian follicles. Estrogen is secreted from the ovaries and then transported in the blood to target tissues containing one or both nuclear estrogen receptor subtypes (ERα and ERβ) (Dahlman-Wright et al. 2006) and plasma membrane receptors, such as G protein-coupled estrogen receptor 1 (GPER1) (Olde and Leeb-Lundberg 2009). Estrogen produces biological effects through genomic and non-genomic mechanisms after binding to ERs in cells (Nilsson et al. 2001; Levin and Hammes 2016). As women enter menopause, the follicles in the ovaries are depleted due to atresia during each menstrual cycle, and the amount of estrogen produced by the ovaries declines. When estrogen levels begin to drop and fluctuate, short-term symptoms such as hot flashes, night sweats, and mood changes frequently arise. The ovaries will eventually stop producing estrogen, and the duration of estrogen deficiency will increase, accelerating the risk of chronic diseases such as osteoporosis, cardiovascular disease, obesity, type 2 diabetes, and urogenital atrophy (Santen et al. 2010; El Khoudary et al. 2020).

Menopausal hormone therapy (MHT) has been used to prevent short-term menopausal symptoms and certain chronic conditions, such as osteoporosis, cardiovascular disease, and Alzheimer’s disease (Santen et al. 2010; Pinkerton 2020). The original MHT regimen which contained only estrogen, was based on a single ligand-single-receptor model in which the estrogen molecule binds to the ligand-binding domain (LBD) of the estrogen receptor (ER) (Brzozowski et al. 1997; Shiau et al. 1998). Estrogen alone therapy was discontinued in women with a uterus because it increases the risk of endometrial cancer (Ziel and Finkle 1975) by activating ERα (Shang 2006). Women with a uterus are prescribed a combination of estrogen and progesterone. This combination works through a dual ligand-dual receptor mechanism where progesterone binds to the progesterone receptor (PR) and estrogen binds to the ER. The progesterone/PR complex effectively blocks the proliferative effects of ERα in the uterus. Although progesterone can prevent endometrial cancer, the Women’s Health Initiative (WHI) trial found that the addition of medroxyprogesterone acetate to conjugated equine estrogens caused a greater risk of breast cancer, heart attack, venous thromboembolism (VTE), and probable dementia compared to estrogen alone (Rossouw et al. 2002; Anderson et al. 2004; Manson et al. 2013). The WHI results provide convincing evidence that progesterone exacerbates some adverse risks caused by estrogen. The risks of MHT have overshadowed its benefits, including reductions in hot flashes, mood changes, vulvar and vaginal atrophy, osteoporosis, fractures, type 2 diabetes, and colon cancer (Manson et al. 2013; Mauvais-Jarvis et al. 2017). After the publication of the WHI results, the number of women using MHT dropped sharply, and clinical recommendations have undergone major changes. MHT is recommended only for the treatment of moderate to severe vasomotor symptoms and vulvar and vaginal atrophy for only 5 years (Martin and Manson 2008; Pinkerton 2020) because a longer treatment time progressively increases the risk of breast cancer, stroke, VTE, and Alzheimer’s disease (Rossouw et al. 2002; Savolainen-Peltonen et al. 2019; Collaborative Group on Hormonal Factors in Breast Cancer 2019). Although considered safe for healthy postmenopausal women up to age 60, short-term treatment reduces the value of MHT, as 5 years of treatment is often insufficient. The median duration of hot flashes and night sweats is 7.4 years, and many postmenopausal women suffer from these symptoms for more than 14 years (Avis et al. 2015). In addition, vulvar and vaginal atrophy is a chronic progressive disease that usually requires long-term estrogen therapy. The recommendation that MHT should not be used for the primary prevention of chronic diseases associated with menopause (US Preventive Services Task Force 2017), such as diabetes, obesity, and cardiovascular disease also significantly reduces the impact of current MHT formulations. Menopausal women will not be able to reap the full benefits of estrogen until safer formulations of MHT are developed that maintain uterine safety without increasing the risk of breast cancer, blood clots, and other adverse effects.

The adverse risk associated with MHT in postmenopausal women is a pathophysiological conundrum. The risk of breast and uterine cancer, cardiovascular disease, VTE, dementia, Alzheimer’s disease, type 2 diabetes (T2DM), and osteoporosis increase with age (D’Agostino et al. 2008; Leening et al. 2014; Reidel et al. 2016; Rojas and Stuckey 2016; Cobin and Goodman 2017; Winters et al. 2017; Scheyer et al. 2018; Woodward 2019). It is not clear why the use of MHT to restore premenopausal sex hormone levels increases the risk of breast cancer, VTE, probable dementia, and Alzheimer’s disease, while reducing the risk of osteoporosis and T2DM (Manson et al. 2013; Mauvais-Jarvis et al. 2017; Savolainen-Peltonen et al. 2019; Collaborative Group on Hormonal Factors in Breast Cancer 2019). In a clinical setting, it is impossible to calculate the actual risk–benefit ratio of MHT for individual women. Therefore, it is imperative to develop safer drugs to minimize the relative risk for each woman who takes MHT.

A new approach to improve the safety of MHT is to combine estrogens with the selective estrogen receptor modulator (SERM) bazedoxifene, instead of progesterone (Komm et al. 2014; Archer et al. 2016). Bazedoxifene prevents estrogen from binding to ERα, thereby preventing the proliferation of cells in the uterus. It is not clear whether bazedoxifene will prevent the proliferation of breast cells by estrogen to reduce the risk of breast cancer. This combination is approved only for short-term treatment of vasomotor symptoms, so it does not broaden the clinical indications or extend the therapeutic window compared to the combination of estrogen and progesterone. Since estrogen/progesterone and estrogen/SERM combinations are only approved for short-term treatment of vasomotor symptoms and vulvovaginal atrophy, there is a clear need to develop safer MHT formulas for long-term treatment of women who have prolonged menopausal symptoms, as well as the prevention of chronic diseases associated with menopause. To develop safer drugs for long-term MHT, alternative classes of drugs need to be discovered that do not operate through the dual ligand-dual receptor mechanism like estrogen and progesterone or by acting as an antagonist that blocks the binding of estrogens to ER as SERMs do. Our goal is to combine estrogen with a drug that has a different mechanism of action than progesterone and SERMs to overcome its adverse effects. In this study, we investigated if 2′,3′,4′-trihydroxychalcone could be an alternative to progesterone and SERMs in MHT by reprogramming the effects of E2 on ERα-mediated gene regulation and breast cancer cell proliferation.

## Materials and methods

### Compounds

2′,3′,4′-trihydroxychalcone and the other chalcones were obtained from Indofine Chemical Company (Hillsborough Township, NJ, USA). The structure of 2′,3′,4′-trihydroxychalcone (Catalog Number: T-501, Lot Number: 93033) was verified by the QB3 NMR Facility at the University of California, Berkeley. ICI 182,780 (ICI), G-15, and rosiglitazone were obtained from Tocris Bioscience (Minneapolis, MN, USA). All other compounds were obtained from Sigma-Aldrich (St. Louis, MO, USA) or Thermo Fisher Scientific (Waltham, MA, USA). The chalcones, steroids, ICI 182,780, tamoxifen, raloxifene, and rosiglitazone were dissolved in ethanol and used at a final concentration of 0.1%.

### Cell culture

Human U2OS cells expressing a tetracycline-regulated ERα (U2OS-ERα) were prepared, characterized, and maintained as previously described (Tee et al. 2004). The cells were maintained in phenol red-free Gibco DMEM/F-12 (Thermo Fisher Scientific, Waltham, MA, USA) supplemented with 5% charcoal–dextran stripped fetal bovine serum (FBS, Gemini Bio Products, West Sacramento, CA, USA), 100 units/mL penicillin and streptomycin, 50 μg/mL Fungizone, and 2 mM of glutamine. To maintain stable transfected cells, 50 μg/mL hygromycin B and 500 μg/mL of zeocin (Invitrogen, Waltham, MA, USA) were included in culture media. MCF-7 breast cancer cells were maintained in phenol red-free DMEM/F-12 supplemented with 10% FBS, 100 units/mL penicillin and streptomycin, 50 μg/mL Fungizone, and 2 mM of glutamine. For experiments, the culture medium was replaced with 5% charcoal–dextran stripped FBS in phenol red-free DMEM/F12.

### Cell transfection and luciferase reporter assay

U2OS cells (wild type) were maintained in 5% charcoal–dextran stripped FBS. The cells were transfected with 3 μg of a plasmid containing the ERE upstream of the minimal thymidine kinase luciferase promoter (ERE-TK-Luc) or 3 copies of the ER regulatory element (Levy et al. 2007) in the NKG2E promoter (NKG2E-TK-Luc) and 1 μg of an ERα expression vector by electroporation as previously described (An et al. 2001). MCF-7 cells were maintained for 3 days in 5% charcoal–dextran stripped FBS prior to transfection by electroporation with 5 μg ERE-TK-Luc. Transfection of other nuclear receptors in U2OS cells was performed by electroporation with 3 μg reporter plasmid and 1 μg of the corresponding nuclear receptor expression vector. The transfected cells were treated with various compounds for 24 h, and then lysed and assayed for luciferase activity using the Luciferase Assay System (Promega, Madison, WI, USA) according to the manufacturer’s protocol. Relative light units (RLU) were measured with a luminometer. Cell-based assays to determine the effects 2′,3′,4′-THC on pregnane X receptor (PXR) and constitutive androstane receptor (CAR1) activation were performed by Puracyp (Carlsbad, CA, USA) using their proprietary reporter cell lines.

### Estrogen receptor binding assay

MCF-7 cells were incubated with 5 nM [^3^H]-E2 (specific activity 87.6 Ci/mmol; PerkinElmer, Waltham, MA, USA) in the presence of increasing concentrations of 2′,3′,4′-THC at 37 °C for 1 h as previously described (Cvoro et al. 2007). After washing the cells with 0.1% bovine serum albumin in phosphate buffered saline (PBS), 100% ethanol was added to the cells. Radioactivity in the samples was measured with a scintillation counter. Specific binding of [^3^H]-E2 was calculated as the difference between total and nonspecific binding in counts per minute.

### RNA isolation and quantitative real-time PCR

Total cellular RNA was extracted using the Aurum Total RNA Mini Kit (Bio-Rad Laboratories, Hercules, CA, USA) following the manufacturer’s protocol. Reverse transcription reactions were performed using the iScript cDNA Synthesis Kit (Bio-Rad, Hercules, CA, USA) with 1 μg of total RNA according to the manufacturer’s protocol. Quantitative reverse transcription-polymerase chain reaction (qRT-PCR) was performed with a Bio-Rad CFX96 Thermal Cycler System using SsoFast EvaGreen Supermix (Bio-Rad, Hercules, CA, USA). The results were analyzed by competitive Ct method (Schmittgen and Livak 2008). The Ct values of specific genes were normalized to the reference gene glyceraldehyde-3-phosphate dehydrogenase (GAPDH) running concurrently to obtain adjusted Ct values (∆Ct). Fold changes were calculated by comparing ∆Ct values from various treatments to control samples. Table S1A shows the primers used for qRT-PCR (Additional file 1).

### Microarray and data analysis

Total cellular RNA was isolated using the Aurum Total RNA Mini Kit (Bio-Rad, Hercules, CA, USA) per the manufacturer's directions. RNA was first quantified with a nanodrop, and then qualitatively evaluated by the Bio-Rad Experion system per the manufacturer’s instructions. Biotin-labeled complementary RNA samples were prepared using 750 ng of total RNA. Biotin-labeled samples were evaluated by both 260/280 absorbance spectrophotometry and capillary electrophoresis. Labeled complementary RNA samples were hybridized overnight to Human Genome HG U133A-2.0 Affymetrix GeneChip arrays (Thermo Fisher Scientific, Waltham, MA, USA). All treatments were done in triplicate with the same batch of microarrays. The data was analyzed as previously described (Tee et al. 2004).

### Western blot analysis

Western blotting was performed as previously described (Pan et al. 2016). In brief, the cells were grown in 6-well tissue culture dishes to reach 80% confluence. The cultured medium was replaced with serum-free DMEM for 24 h before the cells were treated with the compounds. The cells were lysed in radioimmunoprecipitation assay (RIPA) buffer containing the Roche cOmplete protease inhibitor cocktail (Sigma-Aldrich, St. Louis, MO, USA). Total protein concentration of the cell lysate was determined by the Bradford method using Coomassie Plus protein Assay Reagent (Thermo Fisher Scientific, Waltham, MA). Proteins in cell lysates from each sample (15 µg) were separated by SDS-PAGE on a 4–12% Bis–Tris NuPage gel with MOPS running buffer (Thermo Fisher Scientific, Waltham, MA, USA) and then transferred to an Immobilon-P polyvinylidene difluoride (PVDF) membrane (Sigma-Aldrich, St. Louis, MO, USA). The membrane was blocked with 10% nonfat dry milk in tween-tris-buffered saline (TTBS) at room temperature for 1 h. A mouse anti-c-MYC antibody (Takara Bio USA, Inc, Mountain View, CA, USA) was used at 1 µg/ml in 1% nonfat dry milk-TTBS at 4 °C overnight. After washing with TTBS three times, the membrane was incubated with a goat anti-mouse IgG horseradish peroxidase conjugated antibody (Santa Cruz Biotechnology, Dallas, TX, USA) at 1:10,000 dilution in 1% nonfat dry milk-TTBS for 1 h at room temperature. Immunocomplexes on the PVDF membrane were visualized using the ECL Prime Western Blotting Detection Reagent (GE Healthcare, Chicago, IL, USA). The membrane was then washed with Blot Stripping Buffer (Thermo Fisher Scientific, Waltham, MA, USA) and PBS followed by reprobing with a rabbit anti-β-actin IgG (Santa Cruz Biotechnology, Dallas, TX, USA) and then a goat anti-rabbit IgG horseradish peroxidase conjugated antibody. β-actin was visualized with the ECL Prime Western Blotting Detection Reagent. Active MAPK phospho-p44/42 MAPK (Erk 1/2) monoclonal antibody and inactive p44/42 MAPK (Erk 1/2) monoclonal antibodies (Cell Signaling Technology, Danvers, MA, USA) were used as suggested by the manufacturer. For ERα degradation studies, the ER antibody (ab858) was used as recommended by the manufacturer (Abcam, Boston, MA, USA). The protein bands from the scanned x-ray film were quantified using ImageJ software.

### Chromatin immunoprecipitation (ChIP) assays

ChIP assay was performed as previously described (Pan et al. 2016). U2OS-ERα cells were incubated for 24 h with 1 μg/mL of doxycycline to induce ERα in serum-free DMEM/F12 when the cells reached 80% confluence. The cells were then treated with vehicle, E2, 2′,3′,4′-THC and the combination for 2 h. For MCF-7 cells, the cultured media was switched to serum-free DMEM/F12 upon reaching 80% confluence and incubated for 24 h. The cells were then treated with vehicle, E2, 2′,3′,4′-THC or the combination for 1 h. After treatment, 11X formaldehyde solution was added to the culture media and incubated for 15 min at room temperature with shaking. The reaction was quenched with a 1.25 M glycine solution. The cell monolayer was then washed with PBS containing cOmplete Protease Inhibitor Cocktail (Sigma-Aldrich, St. Louis, MO, USA), collected by scraping, and concentrated by centrifugation (2000×*g*, 4 °C for 5 min). The cell pellets were stored at -80 °C. To perform the ChIP assay, the frozen pellets were lysed with buffer containing 0.5% of Triton X-100, 50 mM Tris (pH 7.4), 150 mM sodium chloride, 10 mM ethylenediaminetetraacetic acid, and protease inhibitor cocktail. Cell lysates were centrifuged, and the pellets were resuspended in RIPA buffer. The suspensions were sonicated on ice using a Digital Sonifier and the supernatants were obtained by centrifugation at 14,000 rpm for 10 min at 4 °C. The samples were then diluted with the appropriate amount of RIPA buffer without detergents. Approximately 10% of each diluted sample was used for the input and stored at 4 °C. The samples were incubated with 4 μg/mL of rabbit anti-ERα IgG (sc-544, Santa Cruz Biotechnology, Dallas, TX, USA) or the same concentration of normal rabbit IgG (sc-2025 Santa Cruz Biotechnology, Dallas, TX, USA) at 4 °C overnight with rotation. The immune complexes were then precipitated with Protein G Magnetic Sepharose beads (GE Healthcare, Chicago, IL) for 4 h while rotating at 4 °C. The DNA–protein complexes were then eluted from the magnetic beads with a 1% SDS, 0.1 M sodium bicarbonate solution at 65 °C for 10 min. The bound cross-linked DNA was reversed by incubation at 65 °C overnight. Eluted DNA was purified and concentrated using the ChIP DNA Clean and Concentrator (Zymo Research, Irvine, CA). ERα antibody precipitated DNA was amplified by qRT-PCR with specific primers (Additional file 1: Table S1B) for the *c-MYC* enhancer region (Wang et al. 2011a) or ERE in *KRT19* (Choi et al. 2000). The Ct values from treatments were adjusted using the corresponding input Ct values. The fold changes were obtained by comparison of adjusted Ct values of treatments with control value.

### Cell proliferation assay

MCF-7 cells were plated at a density of 50,000 cells per well in 6-well tissue culture plates in DMEM/F12 supplemented with 5% stripped FBS. The next day the cells were treated with vehicle or E2 in the absence and presence of increasing doses of 2′,3′,4′-THC for 7 days. The cells were then detached with trypsin, neutralized with media containing 5% FBS, and resuspended. Appropriate amounts of cell suspension were placed in ISOTON II diluent (Thermo Fisher Scientific, Waltham, MA, USA) and the cell numbers were then measured using a Coulter Counter (Beckman, Brea, CA, USA).

### Flow cytometry

Flow cytometry was performed based on a previously described method (Pan et al. 2016). Briefly, the cells were plated at a density of 500,000 cells per well in 6-well tissue culture dishes in DMEM/F-12 supplemented with 5% stripped FBS for 48 h. The cultured medium was then replaced by serum-free DMEM/F12 for 24 h. The cells were then treated with vehicle, E2 without or with the indicated amount of 2′,3′,4′-THC in the figure legend for 24 h. The culture medium was then aspirated, and the cells were washed with PBS, detached with trypsin and collected by centrifugation at 1700 rpm for 5 min. The cell pellets were washed with ice cold PBS followed by centrifugation at 1700 rpm for 10 min at room temperature. The cell pellets were resuspended in 500 μL PBS containing 50 μg/mL propidium iodide, 0.1% of triton X-100, 0.1% of sodium citrate, and 10 μg/mL of RNase. The cell suspensions were then analyzed with a BD LSR II Flow Cytometer (BD Biosciences, San Jose, CA, USA) in the Flow Cytometry facility at University of California, Berkeley and the percentage of cells in cell cycle phases were determined by using FlowJo 7.6.5 (FlowJo, LLC, Ashland, OR, USA).

### Statistical analysis

All data are presented as the mean ± SE or SD from at least biological triplicates. The statistical significance of the difference between two groups was assessed by the Student’s *t-*test. For the data sets consisting of more than two groups, the statistical significance of differences among various groups (treatments) were analyzed by one-way analysis of variance (one-way ANOVA) tests or two-way ANOVA as specified in figure legends. All ANOVA tests were followed by Tukey’s or Sidak’s multiple comparisons post hoc tests to analyze the significance of differences between any two different treatment groups or control, as indicated in the figure legend. Statistical analysis and graph plotting were performed using GraphPad Prism version 6 (GraphPad Software Inc., San Diego, CA, USA). The statistical significance for the numbers of asterisks in the figures are *p < 0.05; **p < 0.01, ***p < 0.001, and ****p < 0.0001.

## Results

### 2′,3′,4′-THC acts synergistically with E2 in U2OS cells

**Fig. 1:**
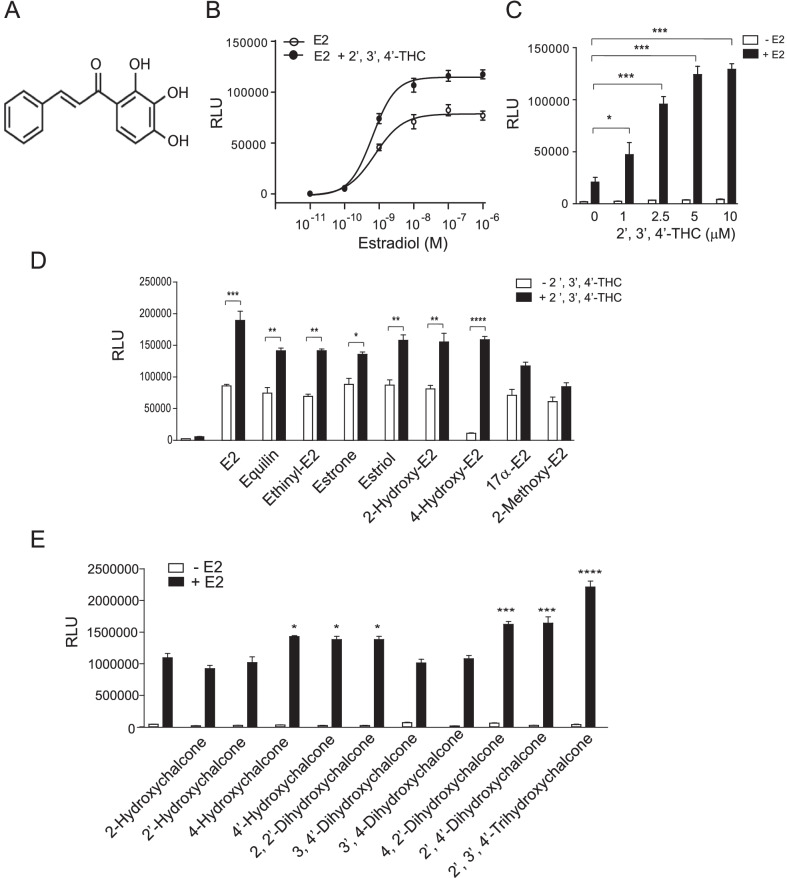
2′,3′,4′-THC synergizes with E2 to induce transcription with ERα. **A** Chemical structure of 2′,3′,4′-THC. **B** U2OS cells cotransfected with ERE-TK-Luc and ERα were treated with increasing concentrations of E2 in the absence and presence of 5 μM 2′,3′,4′-THC for 24 h. The average RLU for the control cells was 2551 and the 2′,3′,4′-THC treated cells was 2650. **C** U2OS cells cotransfected with NKG2E-TK-Luc and ERα were treated with increasing concentrations of 2′,3′,4′-THC in the absence and presence of 10 nM E2 for 24 h. **D** U2OS cells cotransfected with NKG2E-TK-Luc and ERα were treated with 10 nM E2 or 100 nM of the other estrogens in the absence and presence of 5 μM 2′,3′,4′-THC for 24 h. **E** U2OS cells cotransfected with NKG2E-TK-Luc and ERα were treated with 5 μM of each chalcone in the absence or presence of 10 nM E2 for 24 h. Luciferase activity was measured in cellular lysates with a luminometer. RLU is relative light units. Each point in the figures represents the mean of triplicate samples ± SE. **C, D** The asterisks indicate statistical significance between the two groups analyzed by t-test. **E** The asterisks over the bars indicate the significant difference between E2 alone and E2 in combination with the chalcone as analyzed by t-test

Drugs that bind to ERs have two main pharmacological properties. They can be used as agonists to trigger biological responses or as antagonists to block the effects of agonists. E2 and conjugated equine estrogens are the main agonists used in MHT. SERMs have both agonist and antagonistic properties depending on the target tissue (Maximov et al. 2013), although they are mainly used in MHT for their antagonist activity in the uterus. Since both agonist alone and agonist plus antagonist approaches have not produced a MHT formula that is approved for long-term use due to safety concerns, we screened compounds with a similar molecular size to E2 for their ability to produce a synergistic action when combined with E2. We reasoned that if a compound has a synergistic effect with E2, rather than acting as an agonist or antagonist, it may alter the biological actions of ERα to prevent its adverse effects, improving the safety of E2, and possibly lower the pharmacological dose of E2 required to achieve clinical benefits. In a preliminary screening of compounds, we found that a class of natural compounds called chalcones produced a synergistic effect when combined with E2. These screenings resulted in the discovery of 2′,3′,4′-THC (Fig. 1A), which is a small molecule that consists of two aromatic rings separated by a propanone group with a molecular weight (256 Daltons) close to E2 (272 Daltons).

We used U2OS osteosarcoma cells to explore the synergistic action of 2′,3′,4′-THC in more detail, since many studies have used these cells to study the mechanism of action of estrogens. U2OS cells were transfected with ERE-TK-Luc and an expression vector for ERα, and then treated for 24 h with 2′,3′,4′-THC in the absence and presence of increasing concentrations of E2. No significant activation of ERE-TK-Luc occurred with 2′,3′,4′-THC alone, whereas E2 produced a maximal activation of ERE-TK-Luc at 10 nM (Fig. 1B). 2′,3′,4′-THC produced a synergistic activation at all E2 concentrations tested, even at saturating levels (10 nM to 1 μM). We consider the effect of 2′,3′,4′-THC to be a synergistic response because the combination of 2′,3′,4′-THC and E2 produces a greater activation than the sum of the two individual compounds since 2′,3′,4′-THC has no activation effect alone. In a previous study, we found that the NKG2E promoter contains a complex ER regulatory element (Levy et al. 2007) that has the advantage over an ERE because it is activated by both E2 and SERMs in U2OS cells (Levy et al. 2007). Because our goal is to develop a drug that does not act as a SERM it is important to show that 2′,3′,4′-THC does not activate the NKG2E promoter like the SERMs. U2OS cells were transfected with NKG2E-TK-Luc and then treated with increasing concentrations of 2′,3′,4′-THC in the absence and presence of E2. Unlike the SERMs, tamoxifen and raloxifene (Levy et al. 2007), 2′,3′,4′-THC did not activate NKG2E-TK-Luc (Fig. 1C). A synergistic response with E2 was observed at 1 μM 2′,3′,4′-THC and the maximal response occurred at 5 μM. A synergistic effect by 2′,3′,4′-THC was observed with other estrogens, including equilin, ethinyl estradiol, estrone, and estriol, and the E2 metabolites, 2-hydroxyestradiol and 4-hydroxyestradiol (Fig. 1D). No significant synergistic activation was observed with 17α-estradiol and 2-methoxyestradiol. Structure activity relationships with different chalcones were performed with transfection assays using NKG2E-TK-Luc. The synergy was greatest with 2′,3′,4′-THC followed by 4,2′-dihydroxychalcone (DHC) and 2′,4′-DHC (Fig. 1E). These studies indicate that the 2′ and 4′ OH groups of 2′,3′,4′-THC (Fig. 1A) are important for the synergistic effect.

**Fig. 2:**
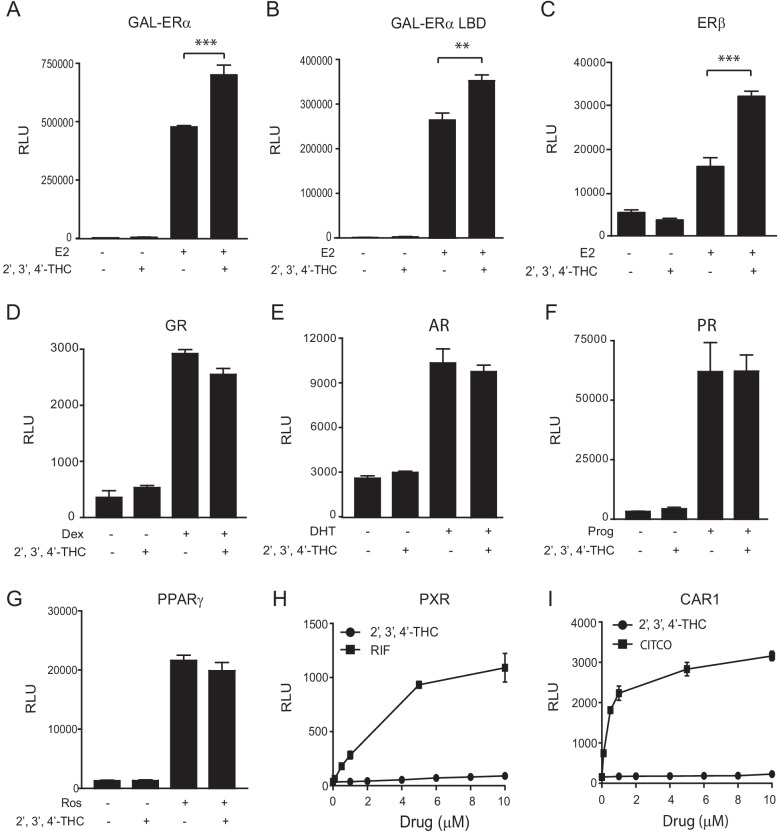
The synergistic effect of 2′,3′,4′-THC is selective for ERs. **A** U2OS cells cotransfected with GAL-TK-Luc and an expression vector for GAL4-DBD-full-length ERα were treated with 10 nM E2 and 5 μM 2′,3′,4′-THC alone or in combination for 24 h. **B** U2OS cells cotransfected with GAL-TK-Luc and an expression vector for GAL4-DBD-ERα LBD were treated with 10 nM E2 and 5 μM 2′,3′,4′-THC alone or in combination for 24 h. **C** U2OS cells cotransfected with ERE-TK-Luc and an expression vector for ERβ were treated with 10 nM E2 and 5 μM 2′,3′,4′-THC alone or in combination for 24 h. **D** U2OS cells cotransfected with GRE-TK-Luc and human GR were treated with 10 nM dexamethasone (Dex) and 5 μM 2′,3′,4′-THC alone or in combination for 24 h. **E** U2OS cells cotransfected with human tyrosine aminotransferase 3 (TAT3)*-*Luc reporter gene and human AR were treated with 10 nM dihydrotestosterone (DHT) and 5 μM 2′,3′,4′-THC alone or in combination for 24 h. **F** U2OS cells cotransfected with TAT3-Luc and human PR-B were treated with 10 nM progesterone (Prog) and 5 μM 2′,3′,4′-THC alone or in combination for 24 h. **G** U2OS cells cotransfected with PPAR-RE-Luc and human PPARγ were treated with 10 μM rosiglitazone (Ros) and 5 μM 2′,3′,4′-THC alone or in combination for 24 h. Luciferase activity was measured in cellular lysates with a luminometer. RLU is relative light units. The data shown are mean of triplicate samples ± SE. **H,** **I** 2′,3′,4′-THC does not alter PXR and CAR1 activity. **H** PXR and **I** CAR1 activity was assayed with Puracyp’s trademark stable reporter cell lines. RIF, Rifampicin is a PXR agonist. CITCO, 6-(4-Chlorophenyl)imidazo[2,1-b][1,3]thiazole-5-carbaldehyde O-(3,4-dichlorobenzyl)oxime is a CAR agonist

To determine the region of ERα that is required for the synergy, U2OS cells were transfected with five copies of the GAL-RE upstream of TK-Luc (GAL-TK-Luc) and a vector that expresses the full-length ERα or ERα LBD fused to the GAL4-DNA binding domain. Synergistic activation of GAL-TK-Luc by the 2′,3′,4′-THC/E2 combination occurred with both the GAL-DBD-full-length ERα (Fig. 2A) and GAL-DBD-ERα LBD (Fig. 2B) demonstrating that the ERα LBD alone can produce a synergistic effect. The synergistic effect of 2′,3′,4′-THC was also observed with ERβ (Fig. 2C), but not with the human glucocorticoid (GR, Fig. 2D), androgen (AR, Fig. 2E), progesterone-B (PR, Fig. 2F), and peroxisome proliferator-activated receptor gamma (PPARγ, Fig. 2G) receptors. In addition to interacting with steroid nuclear receptors, drugs such as 2′,3′,4′-THC might interact with the xenobiotic receptors, PXR and CAR1 that are involved in drug metabolism through modulation of phase I and II enzymes (Willson and Kliewer 2002). These nuclear receptors have been implicated in potential side effects and drug-drug interactions (Wang et al. 2012). To evaluate potential adverse effects or drug-drug interactions by 2′,3′,4′-THC, the effect of 2′,3′,4′-THC on PXR and CAR1 activation was examined. 2′,3′,4′-THC did not alter the activity of PXR (Fig. 2H) or CAR1 (Fig. 2I) at all doses tested, providing preclinical evidence that 2′,3′,4′-THC may be safe for future animal and human studies. The studies showing that 2′,3′,4′-THC does not activate other steroid nuclear and xenobiotic receptors suggest that the synergy is specific for ERα and ERβ. We focused on the effects of 2′,3′,4′-THC on ERα instead of ERβ because ERα mediates the proliferative effects of estrogen on breast and uterine cells, which is a critical adverse action that needs to be overcome by new drugs for MHT.

### The 2′,3′,4′-THC/E2 combination leads to the regulation of reprogrammed genes

**Fig. 3:**
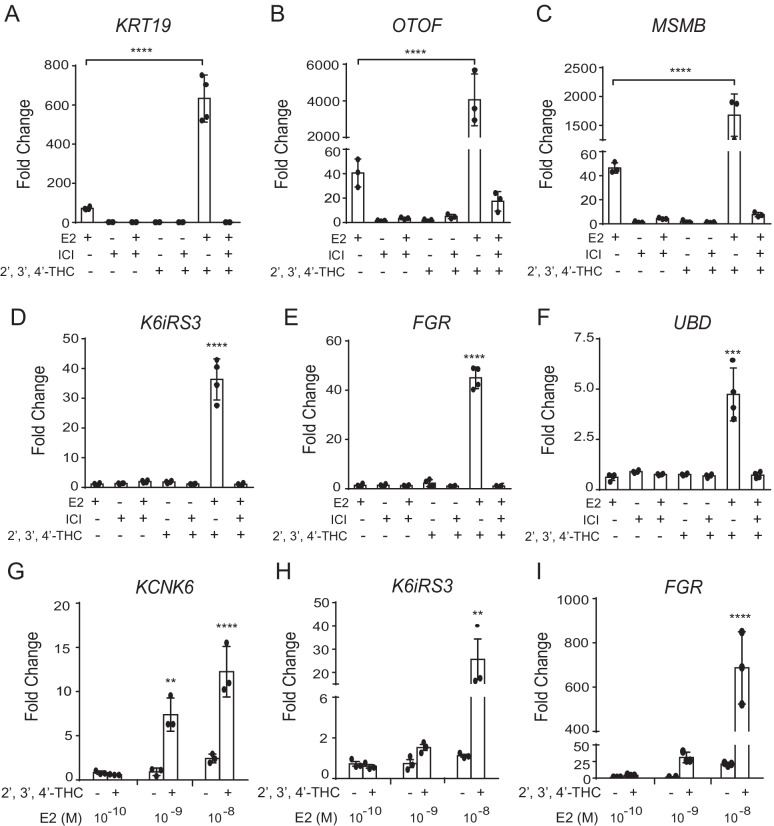
ICI blocks the 2′,3′,4′-THC/E2 combination activation of the synergistic and reprogrammed genes. **A, B, C** Verification of synergistic genes from the microarray in U2OS-ERα cells treated with 10 nM E2, 5 μM 2′,3′,4′-THC, and 1 μM ICI alone or in combination for 24 h. **A** *KRT 19*, **B** *OTOF*, and **C** *MSMB* mRNA levels were measured by qRT-PCR. **D, E, F** Verification of reprogrammed genes from the microarray in U2OS-ERα cells treated with 10 nM E2, 5 μM 2′,3′,4′-THC, and 1 μM ICI alone or in combination for 24 h. **D** *K6iRS3*, **E** *FGR*, and **F** *UBD* mRNA levels were measured by qRT-PCR. **G, H, I** U2OS-ERα cells were treated with increasing doses of E2 in the absence and presence of 5 μM 2′,3′,4′-THC for 24 h. **G** *KCNK6*, **H** *K6iRS3*, and **I** *FGR* mRNA levels were measured by qRT-PCR. GAPDH was used as a reference gene to calculate fold change. The data are mean of triplicate samples ± SD. The statistical significance was determined by one-way ANOVA followed by Tukey’s multiple comparisons post hoc test

In order to determine if 2′,3′,4′-THC alters E2 regulation of endogenous genes, we performed microarray analysis in U2OS-ERα cells. A gene was considered to be regulated by the drug treatment if it was up-regulated or down-regulated by threefold or more, with a p-value ≤ 0.05. E2 alone regulated 756 genes, whereas 2′,3′,4′-THC alone regulated only 31 genes, of which 14 genes were also regulated by E2 and 25 genes were regulated by the 2′,3′,4′-THC/E2 combination (Additional file 2: Table S2). The 2′,3′,4′-THC/E2 combination regulated 1,358 genes (Additional file 2: Table S2). We previously identified E2-regulated genes with microarrays in U2OS-ERα cells and verified some of them by PCR (Tee et al. 2004). Since our objective is to develop a 2′,3′,4′-THC/E2 combination drug for menopausal symptoms, we focused on genes that were regulated by the combination. In comparison to E2 alone, two main classes of regulated genes emerged when 2′,3′,4′-THC was added to E2. Approximately 90 genes showed a synergistic response when cells were treated with the 2′,3′,4′-THC/E2 combination (Additional file 2: Table S2, common genes) as observed in transfection studies with ERE-TK-Luc and NKG2E-TK-Luc reporter genes. The second class of genes is represented by 824 genes that are only regulated by the 2′,3′,4′-THC/E2 combination (Additional file 2: Table S2, reprogrammed genes). These genes were termed reprogrammed genes because they represent a new set of target genes since they are not regulated by E2 or 2′,3′,4′-THC alone. Gene Ontology shows that the molecular pathways regulated by E2 alone (Additional file 3: Fig. S1) were different from those regulated by the 2′,3′,4′-THC/E2 combination (Additional file 4: Fig. S2). Table 1 shows 10 genes as examples of the synergistic and reprogrammed genes from the microarrays. Some of these genes were selected for verification. U2OS-ERα cells were treated with E2 in the absence or in combination with 2′,3′,4′-THC and real-time PCR analysis was performed. Similar to the microarray data (Table 1), E2 alone activated the *KRT19* (Fig. 3A), *OTOF* (Fig. 3B), and *MSMB* (Fig. 3C) genes and a large synergistic effect was observed with 2′,3′,4′-THC that was blocked by ICI. E2 and 2′,3′,4′-THC alone had no effect on reprogrammed genes, *K6iRS3* (Fig. 3D), *FGR* (Fig. 3E), and *UBD* (Fig. 3F). The 2′,3′,4′-THC/E2 combination activated these genes, and ICI blocked the activation. The activation of the reprogrammed genes, *KCNK6* (Fig. 3G), *K6iRS3* (Fig. 3H), and *FGR* (Fig. 3I) by the 2′,3′,4′-THC/E2 combination was dependent on the concentration of E2, suggesting that 2′,3′,4′-THC reprograms the action of E2 to cause it to regulate these genes.

**Table 1:** Representative examples of the synergistic and reprogrammed genes regulated by the 2′,3′,4′-THC/E2 combination from the U2OS-ERα microarrays

Gene name	Gene symbol	Accession	2′,3′,4′- THC	E2	2′,3′,4′- THC + E2
Synergistic genes			Fold change		
Tubulin, alpha 3d	TUBA3D	NM_080386.1	0.9	40.95	485.88
Otoferlin	OTOF	NM_194248.1	1.55	12.19	157.79
Keratin 19	KRT19	NM_002276.3	1.0	17.67	84.18
PRAME family member 4	PRAMEF4	NM_001009611.1	1.17	8.46	48.77
Tyrosine hydroxylase	TH	NM_000360.2	1.16	9.03	45.45
Gamma-aminobutyric acid B receptor, 2	GABBR2	NM_005458.5	1.54	3.86	41.30
Microseminoprotein, beta-	MSMB	NM_002443.2	1.14	3.06	27.84
Neurofilament, heavy polypeptide 200 kDa	NEFH	NM_021076.2	1.03	3.68	26.59
Zinc finger and SCAN domain containing 4	ZSCAN4	NM_152677.1	0.82	5.23	16.21
Methyl-CpG binding domain protein 3-like 2	MBD3L2	NM_144614.2	1.15	5.60	14.49
Reprogrammed genes					
ATP-binding cassette, sub-family A (ABC1), member 3	ABCA3	NM_001089.1	1.24	1.85	53.37
Gardner-Rasheed Feline Sarcoma Viral (V- Fgr) Oncogene Homolog	FGR	NM_001042729.1	1.01	1.07	41.92
Keratin 73	K6IRS3	NM_175068.2	1.19	1.17	37.33
Cytochrome P450, family 4, subfamily F, polypeptide 11	CYP4F11	NM_021187.2	1.12	1.45	29.66
Mucin 1, cell surface associated	MUC1	NM_001044390.1	1.18	1.44	19.14
S100 calcium binding protein A9	S100A9	NM_002965.2	1.26	1.72	18.71
Potassium channel, subfamily K, member 6	KCNK6	NM_004823.1	0.98	0.96	15.88
Cystatin SN	CST1	NM_001898.2	0.93	1.48	15.38
Ubiquitin D	UBD	NM_006398.2	1.00	0.93	11.16
Neuronal guanine nucleotide exchange factor	NGEF	NM_019850.1	1.13	1.70	10.96

The synergistic genes were regulated by E2 alone and the addition of 2′,3′,4′-THC produced a greater activation than the sum of the response by E2 and 2′,3′,4′-THC alone. The reprogrammed genes were not regulated by E2 or 2′,3′,4′-THC alone, but were activated by the 2′,3′,4′-THC/E2 combination. U2OS-ERα cells were treated with 10 nM E2 or 5 μM 2′,3′,4′-THC alone or in combination for 24 h as described in the Methods. The fold change for the gene is the average of triplicate microarrays with a *p-*value ≤ 0.05 for each treatment

### The effect of the 2′,3′,4′-THC/E2 combination on gene expression are ERα dependent

**Fig. 4:**
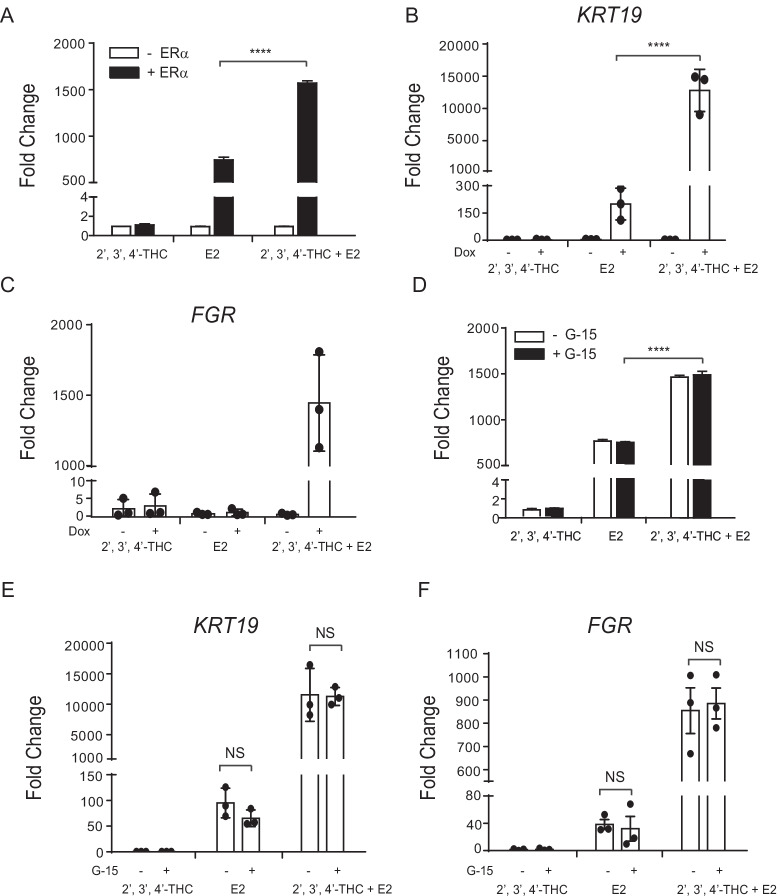
The effect of E2 and the 2′,3′,4′-THC/E2 combination on transcription are ERα-dependent.** A** U2OS cells were transfected with NKG2E-TK-Luc without or with an expression vector for ERα. The cells were treated with 10 nM E2, 5 μM 2′,3′,4′-THC alone or in combination for 24 h and then luciferase activity was measured. **B, C** U2OS-ERα cells were maintained in the absence or presence of 1 µg/ml doxycycline to induce ERα for 24 h and then were treated with 10 nM E2, 5 μM 2′,3′,4′-THC alone or in combination for 24 h. **B** *KRT19* and **C** *FGR* mRNA levels were measured by qRT-PCR. *GAPDH* was used as a reference gene to calculate fold change. **D** The GPER1 antagonist G-15 had no effect on the activation of NKG2E-TK-Luc. U2OS cells were transfected with NKG2E-TK-Luc and an expression vector for ERα. The cells were treated with 10 nM E2, 5 μM 2′,3′,4′-THC alone or in combination for 24 h in the absence and presence of 1 μM G-15, and then luciferase activity was measured. **E, F** U2OS-ERα cells were treated with 10 nM E2, 5 μM 2′,3′,4′-THC alone or in combination for 24 h in the absence and presence of 1 μM G-15. **E** *KRT19* and **F** *FGR* mRNA levels were measured by qRT-PCR. The data are mean of triplicate samples ± SD. The statistical significance was determined by one-way ANOVA followed by Tukey’s multiple comparisons post hoc test

To explore if the synergistic activation of the 2′,3′,4′-THC/E2 combination is ERα dependent, U2OS cells were transfected with NKG2E-TK-Luc in the absence and presence of an expression vector for ERα. No synergistic activation of NKG2E-TK-Luc occurs with E2 and 2′,3′,4′-THC treatment in cells that do not express ERα, whereas a synergistic activation was observed in U2OS cells transfected with ERα (Fig. 4A). In agreement with the transfection studies, the 2′,3′,4′-THC/E2 combination did not activate the synergistic gene, *KRT19* (Fig. 4B) or the reprogrammed gene, *FGR* (Fig. 4C) in U2OS-ERα cells that were not treated with doxycycline (Dox) to induce ERα. To study if the membrane ER, GPER1 was involved in the synergy, U2OS cells were transfected with NKG2E-TK-Luc and ERα and then treated with E2 and 2′,3′,4′-THC in the absence and presence of G-15, which is a selective GPER1 antagonist (Dennis et al. 2009). There was no effect of G-15 on the activation of NKG2E-TK-Luc by E2 or the 2′,3′,4′-THC/E2 combination (Fig. 4D). G-15 did not alter the synergistic activation of the *KRT19* gene (Fig. 4E) or *FGR* induction (Fig. 4F) by the 2′,3′,4′-THC/E2 combination. These results show that the effect of 2′,3′,4′-THC/E2 combination is mediated by ERα.

### 2′,3′,4′-THC does not act like a SERM

**Fig. 5:**
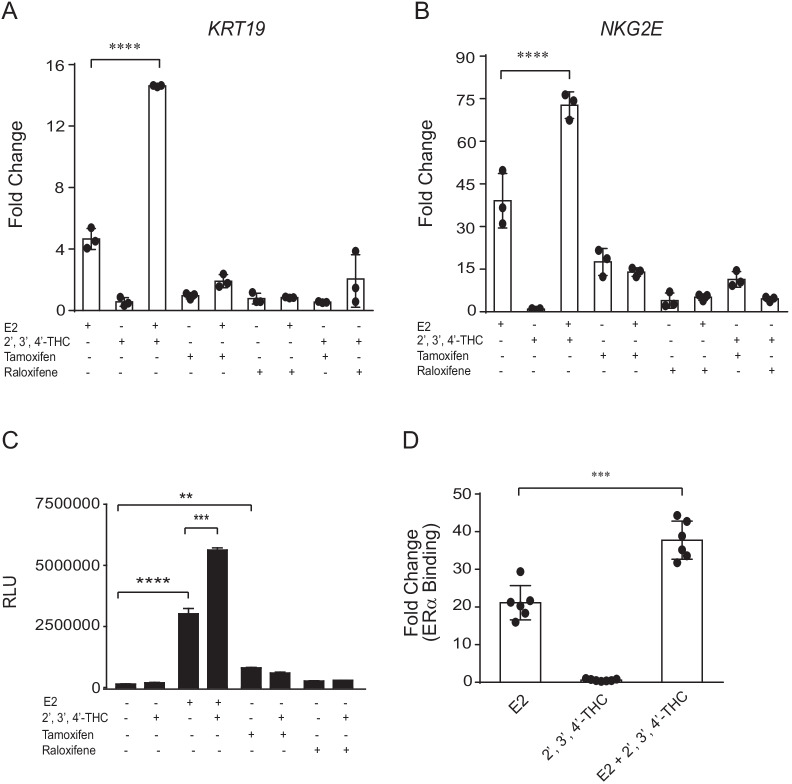
2′,3′,4′-THC acts differently from SERMs on gene transcription**. A** *KRT19* and **B** *NKG2E* gene expression in U2OS-ERα cells treated with 10 nM E2 or 5 μM 2′,3′,4′-THC in the absence and presence of 1 μM tamoxifen or 1 μM raloxifene alone or in combination for 24 h. The mRNA levels were determined by qRT-PCR. **C** U2OS cells cotransfected with ERα and NKG2E-TK-Luc were treated with 10 nM E2, 5 μM 2′,3′,4′-THC, 5 μM tamoxifen, and 1 μM raloxifene alone or in combination for 24 h. Luciferase activities were measured with a luminometer. **D** ERα recruitment to the *KRT19* ERE in U2OS-ERα cells was determined with ChIP assay after the cells were treated with 10 nM E2 and 5 μM 2′,3′,4′-THC alone or in combination for 2 h. The *KRT19* gene was used because the ERE was previously characterized and the ERE in the reprogrammed genes is not known. The data shown are the mean of triplicate samples ± SE. The statistical significance was determined by one-way ANOVA followed by Tukey’s multiple comparisons post hoc test

The crystal structure of ERα shows that SERMs bind to the same binding pocket as E2 (Brzozowski et al. 1997; Shiau et al. 1998). Unlike the SERMs, 2′,3′,4′-THC did not exhibit an antagonist activity on the ERE-TK-Luc or NKG2E-TK-Luc. To compare the activity of 2′,3′,4′-THC to SERMs on endogenous genes, we examined their effects on the expression of the *KRT19* and *NKG2E* genes. E2 induced *KRT19* mRNA expression in U2OS-ERα cells (Fig. 5A). No effect was observed with 2′,3′,4′-THC, tamoxifen, or raloxifene. The 2′,3′,4′-THC/E2 combination produced a synergistic activation of *KRT19*, whereas tamoxifen and raloxifene act as antagonists by blocking the activation by E2 (Fig. 5A). In addition to E2, raloxifene and tamoxifen activated the *NKG2E* gene (Fig. 5B). A synergistic activation of the *NKG2E* gene occurred with the 2′,3′,4′-THC/E2 combination, while tamoxifen and raloxifene antagonized the E2 activation. No synergy or antagonism of the tamoxifen or raloxifene activation of the *NKG2E* gene occurred with 2′,3′,4′-THC (Fig. 5B). Similar results were observed in transfection assays. NKG2E-TK-Luc was activated by E2, tamoxifen, and raloxifene, but 2′,3′,4′-THC produced a synergistic activation only with E2 (Fig. 5C). ChIP shows that the 2′,3′,4′-THC/E2 combination increased the recruitment of ERα compared to E2 alone (Fig. 5D) to a known ERE in the *KRT19* gene (Choi et al. 2000) suggesting that enhanced binding of ERα leads to the synergy. These observations show that 2′,3′,4′-THC does not function as an estrogen or SERM in U2OS cells because it did not act as an agonist or antagonist.

### 2′,3′,4′-THC reprograms the effect of E2 on MCF-7 breast cancer cell proliferation

**Fig. 6:**
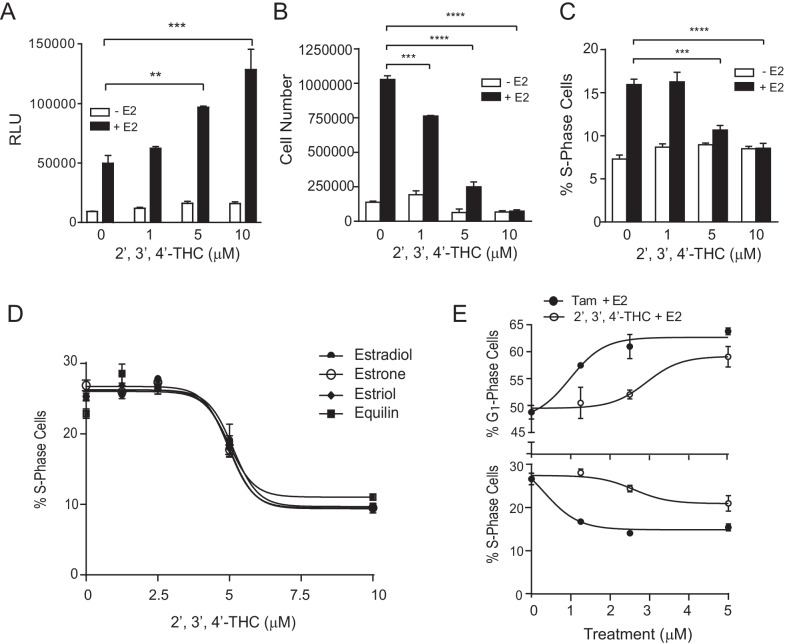
E2-induced MCF-7 breast cancer cell proliferation is blocked by 2′,3′,4′-THC. **A** MCF-7 cells transfected with ERE-TK-Luc were treated with 5 μM 2′,3′,4′-THC and 10 nM E2 alone or in combination for 24 h and then luciferase activity was measured. **B** Growth curve in MCF-7 breast cancer cells was determined with a Coulter counter after the cells were treated with increasing concentrations of 2′,3′,4′-THC without or with 1 nM E2 for 7 days. **C** The percentage of S phase cells was determined by flow cytometry after MCF-7 cells were treated with increasing amounts of 2′,3′,4′-THC without or with 0.1 nM E2 for 24 h. **D** Percentage of S phase cells in the cells treated with increasing concentrations of 2′,3′,4′-THC in the presence of 0.1 nM E2 or 1 nM estrone, estriol, or equilin for 24 h. **E** Percentages of S and G_1_ phase cells in the cells treated with increasing amount of tamoxifen (Tam) or 2′,3′,4′-THC in the presence of 0.1 nM E2 for 24 h were determined by flow cytometry. The error bars are means ± SE. The differences among various treatments were analyzed with two-way ANOVA followed by Sidak’s multiple comparisons post hoc test. The asterisks over the bars indicate the significant difference between E2 alone and E2 in combination with increasing amounts of 2′,3′,4′-THC

Estrogens are known to increase breast cancer risk by activating ERα. Thus, we assessed the effects of 2′,3′,4′-THC alone and in combination with E2 using MCF-7 breast cancer cells. Similar to U2OS cells, 2′,3′,4′-THC produced a dose-dependent synergistic activation of E2 stimulation of ERE-TK-Luc in MCF-7 cells (Fig. 6A). E2 expectedly increased MCF-7 cell proliferation, whereas 2′,3′,4′-THC did not cause proliferation at concentrations from 1 to 10 μM (Fig. 6B). 2′,3′,4′-THC blocked the proliferation induced by E2 in a dose-dependent manner (Fig. 6B). It is known that estrogen causes the proliferation of breast cancer cells by stimulating the progression of cells from the G_1_ phase to the S phase (Foster et al. 2001). As measured by flow cytometry, 2′,3′,4′-THC dose-dependently blocked the E2-induced G_1_ phase cells entry into the S phase (Fig. 6C). This finding is consistent with a G_1_ arrest. 2′,3′,4′-THC also inhibited the percentage of cells entering the S phase when stimulated by 1 nM estrone, estriol, and equilin (IC_50_ = 5 μM) similar to 0.1 nM E2 (Fig. 6D). Tamoxifen caused a G_1_ cell cycle arrest (Fig. 6E) at doses only 3- to eightfold lower than 2′,3′,4′-THC (G_1_ phase, EC_50_ = 0.32 μM vs 2.9 μM; S phase IC_50_ = 0.98 μM vs 2.6 μM).

**Fig. 7:**
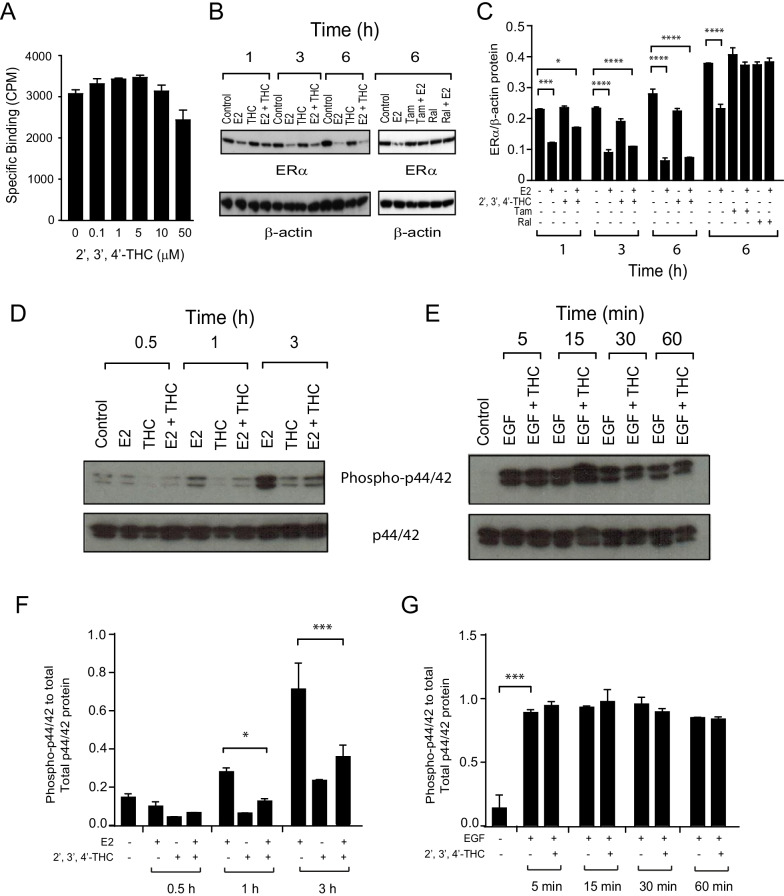
Effects of 2′,3′,4′-THC on ERα degradation and MAPK activity. **A** 2′,3′,4′-THC does not compete for [^3^H]-E2 binding in MCF-7 breast cancer cells. Competitive binding of 2′,3′,4′-THC in the cells treated with 5 nM [^3^H]-E2 and increasing doses of 2′,3′,4′-THC for 1 h at 37 °C. Specific [^3^H]-E2 binding was measured with a scintillation counter and calculated by subtracting non-specific binding from total binding. The error bars are mean ± SE. **B, C** 2′,3′,4′-THC does not alter ERα degradation, whereas SERMs prevent ERα degradation in MCF-7 cells. **B** Western blot of MCF-7 cells treated with 10 nM E2 in the absence and presence of 5 μM 2′,3′,4′-THC, 10 μM tamoxifen (Tam), or 1 μM raloxifene (Ral) for the indicated time and then ERα and β-actin protein levels were determined. **C** Quantification of ERα and β-actin protein levels. The error bars are means ± SE. The differences among various treatments were analyzed with one-way ANOVA. The asterisk over the bars indicates the significant difference between E2 alone and E2 in combination with 2′,3′,4′-THC, Tam, or Ral at various times. **D** 2′,3′,4′-THC inhibits E2 activation of MAPK. Active phospho-p44/42 MAPK (top panel) and inactive p44/42 MAPK (bottom panel) were measured by western blotting after the cells were treated with 10 nM E2, 5 μM 2′,3′,4′-THC alone or in combination for various times. **E** 2′,3′,4′-THC does not alter EGF activation of MAPK. Active phospho-p44/42 MAPK (top panel) and inactive p44/42 MAPK (bottom panel) were measured by western blotting after the cells were treated with 100 ng/ml EGF alone or in combination with 2′,3′,4′-THC for various times. **F** Quantification of phospho-p44/42 and p44/42 protein levels in E2-treated cells. The asterisk over the bars indicates the significant difference between E2 alone and E2 in combination with 2′,3′,4′-THC. **G** Quantification of phospho-p44/42 and p44/42 protein levels in EGF-treated cells. Quantification of protein levels of western blots was done by ImageJ**.** The asterisks indicate a significant difference between the control and EGF-treated cells

Even though 2′,3′,4′-THC did not exhibit antagonistic activity in U2OS cells, the most straightforward explanation for why it inhibits cell proliferation and causes a G_1_ cell cycle arrest in MCF-7 cells is that it prevents E2 binding to ERα, similar to SERMs (Coezy et al. 1982). Since no radiolabeled 2′,3′,4′-THC is available, we performed competition binding studies to determine if 2′,3′,4′-THC competes for [^3^H]-E2 binding sites in MCF-7 cells. No competition of [^3^H]-E2 binding occurred until 50 μM 2′,3′,4′-THC (Fig. 7A). This concentration is 50 times higher than the concentration required for synergy (Fig. 1C) and the antiproliferative effect (Fig. 6B). These findings demonstrate that 2′,3′,4′-THC does not compete with [^3^H]-E2 at active concentrations. To further evaluate the binding of 2′,3′,4′-THC to ERα, we examined the effects of 2′,3′,4′-THC on ERα degradation, since E2 enhances ERα degradation (Nawaz et al. 1999). As expected, E2 produced a time-dependent loss of ERα (Fig. 7B). No change in ERα levels was observed with 2′,3′,4′-THC alone, and it did not block degradation induced by E2 (Fig. 7C). As with 2′,3′,4′-THC, tamoxifen and raloxifene did not cause ERα degradation on their own, but they blocked ERα degradation caused by E2 (Fig. 7B, C). These findings suggest that 2′,3′,4′-THC does not bind to the E2 binding pocket like the SERMs.

### 2′,3′,4′-THC inhibits nongenomic and genomic effects of E2 in MCF-7 cells

**Fig. 8:**
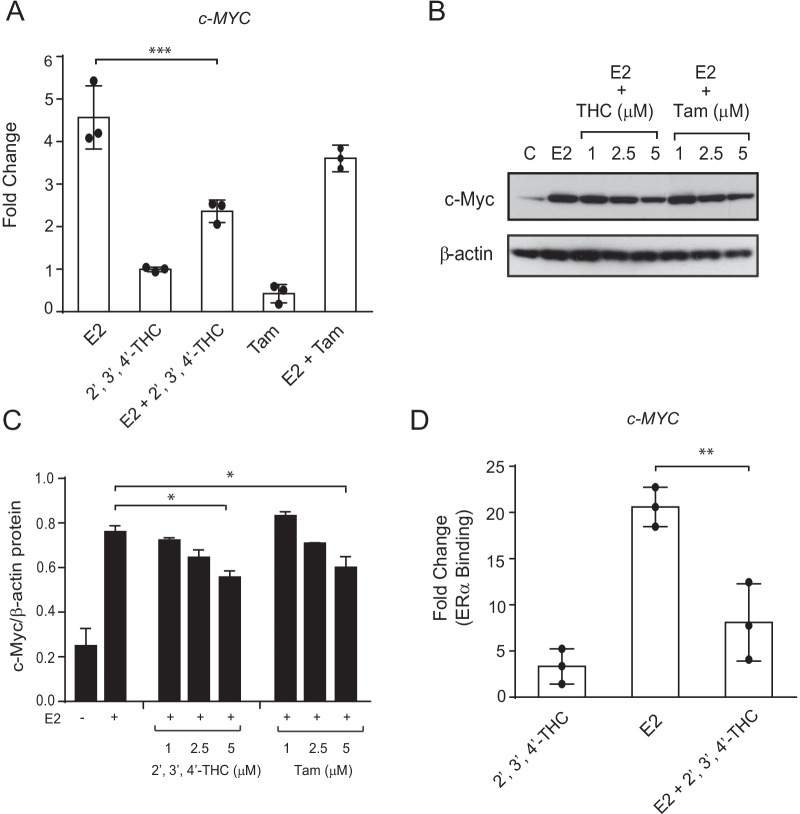
2′,3′,4′-THC inhibits E2 activation of the *c-MYC* gene. **A** MCF-7 cells were treated with 10 nM E2, 5 μM 2′,3′,4′-THC, and 5 μM Tam alone or in combination for 2 h and the levels of *c-MYC* mRNA were measured by qRT-PCR. The error bars are means ± SE. **B** 2′,3′,4′-THC inhibits E2 stimulation of c-MYC protein levels. The cells were treated with 10 nM E2 alone or in combination with increasing doses of 2′,3′,4′-THC (THC) or Tam for 3 h and c-MYC was determined by western blotting. **C** Quantification of c-MYC protein levels in cells treated with E2 and 2′,3′,4′-THC or Tam. **D** 2′,3′,4′-THC inhibits E2-induced ERα recruitment to *c-MYC* enhancer region. The cells were treated with 10 nM E2 without or with 5 μM 2′,3′,4′-THC for 1 h and ERα binding was determined by ChIP assay. Each bar represents the mean of triplicate samples ± SE. Asterisks over bars show the significance between E2 alone and various treatments determined by one-way ANOVA followed by Tukey's multiple comparisons post hoc test

We next investigated the mechanism for the antiproliferative effect of 2′,3′,4′-THC. In addition to their well-characterized genomic effects, estrogenic compounds are also known to have non-genomic effects that promote cell proliferation. E2 activates the MAPK/ERK pathway, resulting in downstream signaling that stimulates cell proliferation (Improta-Brears et al. 1999; Zhang and Liu 2002; Levin 2005). To determine the effect of 2′,3′,4′-THC on the MAPK pathway, MCF-7 cells were treated with E2 in the absence and presence of 2′,3′,4′-THC and the levels of active, phosphorylated p44/p42 MAPK (Erk1/Erk2) were determined by western blotting. A time-dependent increase in phosphorylated p44/p42 MAPK by E2 was inhibited by 2′,3′,4′-THC (Fig. 7D, F). In contrast, 2′,3′,4′-THC had no effect on the phosphorylation of MAPK induced by epidermal growth factor (EGF) (Fig. 7E, G), demonstrating that 2′,3′,4′-THC acts selectively on the ER pathway. The induction of the *c-MYC* gene by E2 is essential for breast cancer cell proliferation (Foster et al. 2001). 2′,3′,4′-THC inhibited the E2 increase of *c-MYC* mRNA (Fig. 8A). The E2-induction of c-MYC protein was reduced by 2′,3′,4′-THC to the same extent as tamoxifen at the same dosages (Fig. 8B, C). ChIP was performed to determine the effect of 2′,3′,4′-THC on ERα recruitment to the *c-MYC* enhancer, which contains an ERα binding site (Shang and Brown 2002; Wang et al. 2011a). E2 caused a significant recruitment of ERα to the *c-MYC* enhancer by 1 h (Fig. 8D), which was inhibited by 2′,3′,4′-THC. This finding suggests that 2′,3′,4′-THC blocks the induction of the c-*MYC* gene by preventing the binding of ERα to the enhancer.

## Discussion

To improve the safety of MHT, we sought to identify drugs that could modulate ERα activity through mechanisms that differ from estrogens and SERMs. We demonstrated that 2′,3′,4′-THC behaves differently from E2 and tamoxifen. Transfection studies with 2′,3′,4′-THC found that it was not an agonist or antagonist, as it did not activate reporter genes like E2 or block the effects of E2 as SERMs. Instead, 2′,3′,4′-THC produces a synergistic activation of ERE-TK-Luc and NKG2E-TK-Luc when E2 is present. Microarray analysis and qRT-PCR showed that 2′,3′,4′-THC alone had little or no agonist activity or antagonism of E2 effects on gene regulation in U2OS-ERα cells. The combination of E2 and 2′,3′,4′-THC regulates two main classes of genes. One class of genes was synergistically activated by the 2′,3′,4′-THC/E2 combination like ERE-TK-Luc and NKG2E-TK-Luc. The other class of genes were termed reprogrammed genes because they are not regulated by ERα unless 2′,3′,4′-THC is added to physiological concentrations of E2. Although it is likely that many of these reprogrammed genes are direct targets of ERα, other genes could be regulated indirectly by gene products induced by the 2′,3′,4′-THC/E2 combination. Our transfection and gene expression studies in the absence of ERα and the presence of the GPER1 antagonist G-15 and data that ICI blocks synergistic activation and the induction of reprogrammed genes by the 2′,3′,4′-THC/E2 combination demonstrate that its effect is mediated by ERα.

Since ERα promotes the proliferation of breast cancer cells in response to E2 (Ali and Coombes 2000), it would appear that the synergistic effect, the absence of antagonism, and the induction of some reprogrammed genes observed in U2OS cells could be harmful. However, our growth curves found that 2′,3′,4′-THC blocks the proliferative response of E2 in MCF-7 cells. The findings that 2′,3′,4′-THC inhibits ERα binding to the c-*MYC* gene, and c-*MYC* mRNA and protein levels suggest that it exerts antiproliferative effects by preventing cells from entering S phase from G_1_, as c-MYC is essential for E2-mediated transition from G_1_ to the S phase (Foster et al. 2001). This suggestion is consistent with flow cytometry data that shows 2′,3′,4′-THC causes a G_1_ arrest. It is also possible that 2′,3′,4′-THC treatment reduces cell number by promoting apoptosis of MCF-7 cells. The antiproliferative effect of 2′,3′,4′-THC is not mediated by ERβ since our MCF-7 cells do not express ERβ (Paruthiyil et al. 2004). While tamoxifen and 2′,3′,4′-THC both cause a G_1_ cell cycle arrest at similar doses, several observations suggest that the mechanism of 2′,3′,4′-THC is not the same as SERMs. First, 2′,3′,4′-THC does not bind to the E2 binding site in ERα because it did not compete with [^3^H]-E2 at active concentrations in MCF-7 cells like tamoxifen (Coezy et al. 1982). Second, tamoxifen increases the binding of ERα to the *c-MYC* gene in MCF-7 cells (Shang and Brown 2002), whereas 2′,3′,4′-THC inhibits E2 recruitment of ERα as demonstrated by the ChIP data. Third, the E2-induced degradation of ERα was blocked by tamoxifen and raloxifene, but not by 2′,3′,4′-THC. The findings that 2′,3′,4′-THC does not block the E2 activation of reporter and endogenous genes and ERα degradation suggest that 2′,3′,4′-THC does not block MCF-7 cell proliferation by the classic antagonistic activity of SERMs that involves binding to the E2 pocket (Brzozowski et al. 1997; Shiau et al. 1998). Our studies suggest that the antiproliferative effect in MCF-7 cells is due to a reprogramming mechanism whereby 2′,3′,4′-THC switches a stimulatory action of E2 on MAPK activity and *c-MYC* transcription to an inhibitory action. We refer to 2′,3′,4′-THC as an ERα reprogramming compound instead of a SERM, since it does not inhibit cell proliferation by binding to the E2 binding site.

A major question raised by our findings is how does 2′,3′,4′-THC reprogram the action of E2 on gene regulation and cell proliferation. One possibility is that E2 and 2′,3′,4′-THC form a heteroligand with E2 binding to one subunit and 2′,3′,4′-THC binding to the E2 binding site on the other subunit of ERα, as described with cotreatment with E2 and SERMs using mutant and chimera ERs (Liu et al. 2013). A heteroligand model is unlikely because 2′,3′,4′-THC did not compete with [^3^H]-E2 binding at concentrations that were biologically active, suggesting that 2′,3′,4′-THC does not bind to the same site as E2 on ERα. It is conceivable that the reprogramming action occurs from the binding of 2′,3′,4′-THC to a secondary site in ERα as a coligand. In a coligand model when E2 binds to its binding pocket, it creates a secondary site that 2′,3′,4′-THC binds to forming a ternary complex consisting of E2, 2′,3′,4′-THC, and ERα. Since the structure of the ternary complex should be different from the binary E2 and ERα complex, it might recognize different regulatory elements that are present in the reprogrammed genes. The formation of the ternary complex and change in ERα conformation could be a potential mechanism whereby 2′,3′,4′-THC inhibits the binding of ERα to the *c-MYC* gene as shown by ChIP. The observation that the size of the ERα binding cavity is nearly two times larger than the molecular volume for E2 (Brzozowski et al. 1997), suggests that two small ligands, such as E2 and 2′,3′,4′-THC can occupy the pocket at the same time. It is also possible that a secondary site is located outside the E2 binding pocket. A coligand model is consistent with the reporter gene data that showed that even when cells are treated with saturating levels of E2 that should occupy all the E2 binding sites, 2′,3′,4′-THC still produces a synergistic effect, possibly by binding to a secondary site on ERα. Future x-ray crystallography studies will need to be done to determine if the reprogramming action of 2′,3′,4′-THC results from its binding to a secondary site on ERα concurrently with E2. Another possibility is that 2′,3′,4′-THC regulates cellular and molecular pathways such as a cell signaling pathway, which could alter the activity of ERα by causing post-translational modifications like phosphorylation (Lannigan 2003), rather than binding directly to ERα. 2′,3′,4′-THC could also reprogram the actions of E2 by binding to coregulatory proteins or transcription factors that interact with ERα (Lonard et al. 2007). A variety of small molecules have been identified that bind to and modulate the activity of coregulatory proteins (Szwarc et al. 2015). The natural polyphenol gossypol directly binds to the steroid receptor coactivators SRC-1 and SRC-3 to promote their degradation (Wang et al. 2011b). While these findings raise the possibility that coregulatory proteins could be a target for 2′,3′,4′-THC, this seems unlikely unless it interacts with an ER-specific coregulatory protein because 2′,3′,4′-THC did not cause synergy with other nuclear receptors, including GR, AR, PR-B, and PPARγ.

Different ligands bind to the same binding pocket of ER to create unique conformations, which leads to the recruitment of distinct coregulatory proteins to alter gene expression profiles (Paige et al. 1999; Nettles et al. 2007; McDonnell and Wardell 2010). A major pharmaceutical strategy to overcome drug side effects is to design and synthesize selective nuclear receptor modulators that bind to the same binding pocket as the cognate ligand to produce distinct conformations and different clinical responses. Another possible approach for drug discovery is to explore ER and other nuclear receptors for secondary binding sites for coligands. Secondary binding sites have been reported to exist for ERs and other nuclear receptors (Kojetinet et al. 2008). It has been suggested that antiestrogens may exert antagonist activity through an allosteric secondary site since ICI increased [^3^H]-E2 binding rather than blocking it in a yeast system (Dudley et al. 2000), which would be expected if it binds to the E2 binding site. A second binding site on the ER is supported by the crystal structure of ER, which showed that 4-hydroxytamoxifen binds at a site outside the E2 binding pocket (Wang et al. 2006). A secondary negative allosteric ligand binding site for 27-hydroxycholesterol has been reported for ERβ based on the observation that it antagonized the activation of E2 on an ERE in reporter assays, but only partially competed with [^3^H]-E2 binding (Starkey et al. 2018). It has been suggested that the antagonism is due to conformational changes caused by the binding of 27-hydroxycholesterol to a secondary site in the ERβ, thereby reducing the binding affinity of E2. If a secondary binding site exists for 2′,3′,4′-THC on ERα, it is conceivable that it overlaps with one that also interacts with antiestrogens or other ligands, even though our studies suggest that the antiproliferative effects of 2′,3′,4′-THC on MCF-7 cells occur through a different mechanism than tamoxifen. Compounds have been identified that bind to a secondary site on membrane receptors that produce positive or negative allosteric effects (Sieghart 2015; Dopart et al. 2018). If nuclear receptors have secondary allosteric ligand binding sites, they may become potential targets for drugs.

Estrogens have been used successfully in MHT to prevent menopausal symptoms and osteoporosis. However, they can produce serious adverse effects, such as breast cancer, endometrial cancer, and blood clots. The pharmacological effects of drugs, such as MHT, vary depending on the individual who uses them (McLean and Le Couteur 2004). At prescribed therapeutic doses, clinical benefits, toxicity, and side effects are rarely universally manifested. Differences in benefits and risks of drugs are often elucidated in clinical trials or in post-marketing pharmacovigilance observations. Genetic and metabolic studies have been used to investigate the different individual responses to pharmacological interventions by detecting mutations in specific target genes or metabolic enzymes in order to identify individuals or groups who have such differences (Spear et al. 2001; Mirsadeghi and Larijani 2017). The risks and benefits associated with combining two or more pharmacological interventions, which are commonly prescribed in clinical practice, are studied through preclinical and clinical drug-drug interaction studies geared towards determining clashes in metabolic enzymes that result in hyperactivation or inhibition of one of the drugs (Benet et al. 2019). Our study showing that 2′,3′,4′-THC does not activate PXR and CAR1 provides preliminary preclinical data suggesting that it may be safe in women.

2′,3′,4′-THC is a member of the diverse naturally occurring flavonoid/chalcone compounds that are found in many plant species commonly consumed by humans in the diet. We showed that there is a significant alteration of the ligand receptor transcriptional outcome of 2′,3′,4′-THC/E2 compared to E2 alone and the combination inhibits MCF-7 breast cancer cell proliferation. The discovery of reprogramming compounds can open a new field of research, which may lead to a greater understanding of the different pharmacological outcomes that are commonly observed in patients. For example, it is not clear why some women develop breast cancer after MHT, but it is conceivable that the presence of reprogramming compounds in the diet may affect the breast cancer outcome of MHT. Natural reprogramming compounds, such as 2′,3′,4′-THC can also be used as chemical scaffolds to design synthetic analogs to enhance selectivity, potency, and bioavailability to improve efficacy and safety. Considering that E2 interacts with many tissues and functions in major physiological processes related to female growth, development, reproduction, and health, a reprogramming drug could have profound implications for research areas related to women’s health.

## Conclusion

Our study shows that 2′,3′,4′-THC may represent a new class of ERα modulators that we have termed a reprogramming compound. 2′,3′,4′-THC exhibits several properties that define their reprogramming action and differentiate them from SERMs. First, it does not have any significant ERα agonist activity alone or antagonistic activity on gene regulation in the presence of E2. Second, it causes a synergistic activation of E2-regulated genes or changes the type of genes regulated by E2. Third, it does not bind to the E2 and SERM binding pocket in ERα to prevent cell proliferation. Our goal is to combine E2 with a reprogramming drug as an alternative to current MHT regimens to block the adverse effects of E2 without interfering with its beneficial effects by interacting with a secondary binding site on ERα or by acting on a non-ER-mediated cellular or molecular pathway that alters ERα activity. The next steps will be to investigate if 2′,3′,4′-THC reprograms ERα responses and evaluate the safety and efficacy of the 2′,3′,4′-THC or analogs of 2′,3′,4′-THC in combination with estrogens in preclinical animal models.

## Supplementary Information

**Additional file 1: Table S1.** A Primers sequence for RT-PCR. B Primers sequence for ChIP.

**Additional file 2: Table S2.** Microarray data.

**Additional file 3: Figure S1.** Gene Ontology (GO) analysis of the molecular function of genes. GO analysis shows that the molecular pathways regulated by E2 alone compared to control cells.

**Additional file 4: Figure S2.** Gene Ontology (GO) analysis of the molecular function of genes. GO analysis shows that the molecular pathways regulated by the 2′,3′,4′-THC/E2 combination compared to E2 alone treated cells. The genes in green (cellular component) and blue (molecular function) bars were new, since they were regulated only by the combination.

## Data Availability

The analyzed microarray data generated are available in the additional material. Other data are available from the corresponding author on request.

## Abbreviations

ANOVA: Analysis of variance
AR: Androgen receptor
CAR: Constitutive androstane receptor
ChIP: Chromatin immunoprecipitation
Ct: Cycle threshold
DBD: DNA binding domain
Dex: Dexamethasone
DHC: Dihydroxychalcone
DHT: Dihydrotestosterone
DMEM: Dulbecco’s Modified Eagle Medium
Dox: Doxycycline
E2: 17β-Estradiol
ECL: Enhanced chemiluminescence
EGF: Epidermal growth factor
ER: Estrogen receptor
ERE: Estrogen response element
ERK: Extracellular signal-regulated kinase
FBS: Fetal bovine serum
GAL-RE: GAL-response element
GPER1: G protein-coupled estrogen receptor 1
GR: Glucocorticoid receptor
ICI: Imperial Chemical Industries 182,780
KRT: Keratin
LBD: Ligand binding domain
Luc: Luciferase
MAPK: Mitogen-activated protein kinase
MHT: Menopausal hormone therapy
MSMB: Microseminoprotein beta
OTOF: Otoferlin
PBS: Phosphate-buffered saline
PCR: Polymerase chain reaction
PPAR: Peroxisome proliferator-activated receptor
PR: Progesterone receptor
Prog: Progesterone
PVDF: Polyvinylidene fluoride
PXR: Pregnane X receptor
Ral: Raloxifene
RIPA: Radioimmunoprecipitation assay
RLU: Relative light units
Ros: Rosiglitazone
qRT-PCR: Quantitative reverse transcription-polymerase chain reaction
SDS-PAGE: Sodium dodecyl sulfate–polyacrylamide gel electrophoresis
SERM: Selective estrogen receptor modulator
Tam: Tamoxifen
TAT3: Tyrosine aminotransferase 3
THC: Trihydroxychalcone
Tk: Thymidine kinase
TTBS: Tween tris-buffered saline
T2DM: Type 2 diabetes mellitus
VTE: Venous thromboembolism
