# Supplementary material for: 2′,3′,4′-Trihydroxychalcone changes estrogen receptor α regulation of genes and breast cancer cell proliferation by a reprogramming mechanism
Source: Mol Med. 2022 Apr 25;28:44. doi: 10.1186/s10020-022-00470-z (PMC9036729; doi:10.1186/s10020-022-00470-z)
Supplement: Supplementary file 1 — Additional file 1: Table S1. A Primers sequence for RT-PCR. B Primers sequence for ChIP. [file 10020_2022_470_MOESM1_ESM.pdf]

Table S1A Primers sequence for RT-PCR

Gene	Forward	Reverse
MSMB	GTGATCTTTGCCACCTTCGT	CGTAGCAAGTGCATGTCTCA
OTOF	CAAAGACGGCAAAGTGGACG	GGCTTCCTCTGACCGTTCT
KCNK6	GCCCGTCTCTGAGCCTTGATTTCCT	AGGCTGGATTGGGCCTAGTCCC
NKG2E	GCCAGCATTTTACCTTCCTCA	AACATGATGAAACCCCGTCTA
K6iRS3	ACAGGGGCTGGCTTTGGATTCAG	GGCAGACTACTGGGAAATGGGCTG
UBD	TGTGGAGTCAGGTGATGAGG	CTGCCATCATCTTCCCATCT
KRT19	TCGCCAAGATCCTG	GCCTCCGTTTCTGC
FGR	CAACCCTCTCTGGCGGTGGC	GCTTGGGGCCAGAGCGGATG
c-MYC	GGAAAACCAGCAGCCTCCCGC	ACGGCTGCACCGAGTCGTAG
GAPDH	CGATGCTGGCGCTGAGTACGT	CCTGCAAATGACCCCCAGCCTTC

Table S1B Primers sequence for ChIP

Gene	Forward	Reverse
KRT19	TCCAGCCTGGGTGACAGAGC	TCCAAGTTCACCCCAACCTGA
c-MYC	ACAAGGATGCGGTTTGTCA	CGCCTACCATTTTCTTTTGC
