# Supplementary figures and images for: 2′,3′,4′-Trihydroxychalcone changes estrogen receptor α regulation of genes and breast cancer cell proliferation by a reprogramming mechanism
Source: Mol Med. 2022 Apr 25;28:44. doi: 10.1186/s10020-022-00470-z (PMC9036729; doi:10.1186/s10020-022-00470-z)

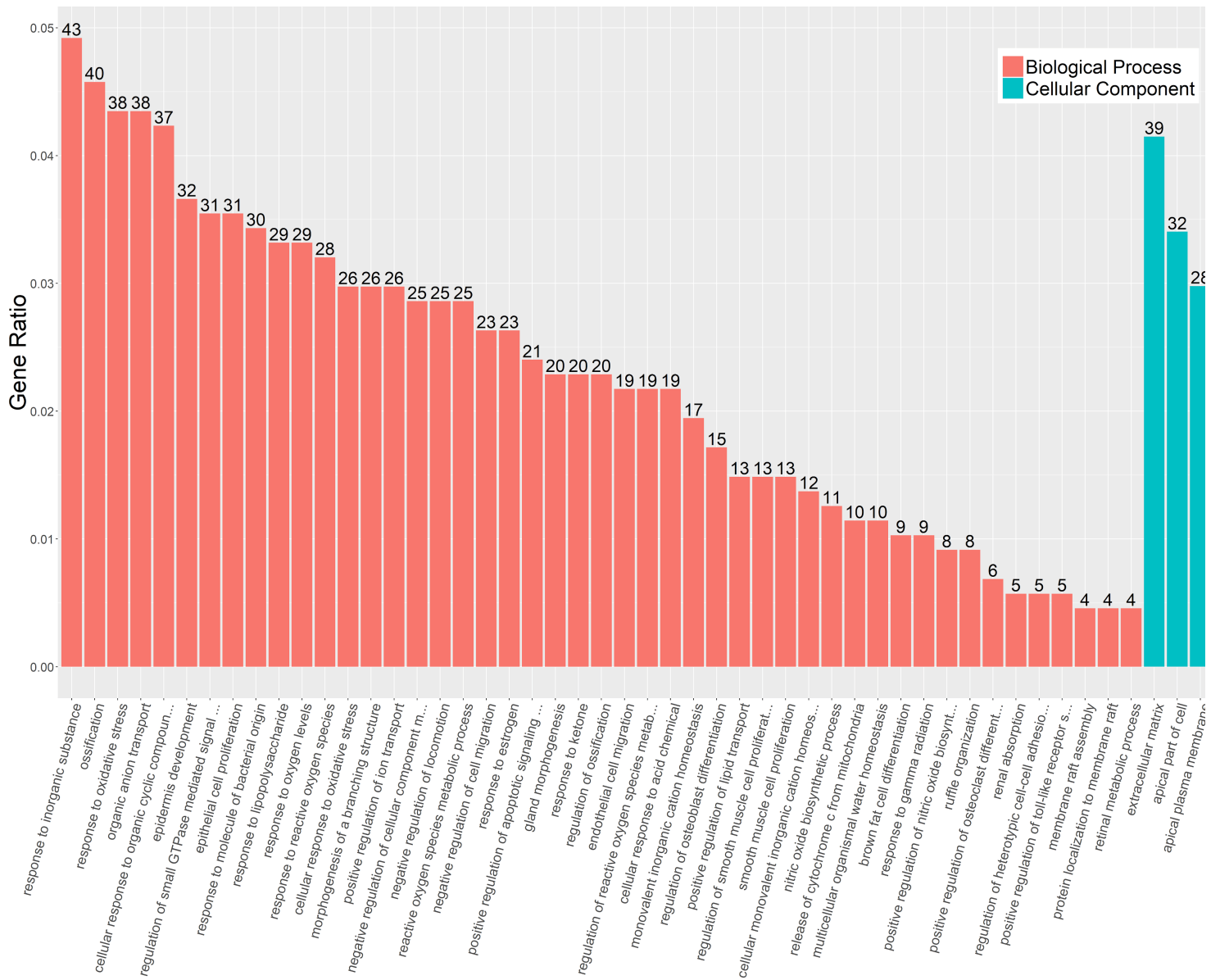

Supplement: Supplementary file 3 — Additional file 3: Figure S1. Gene Ontology (GO) analysis of the molecular function of genes. GO analysis shows that the molecular pathways regulated by E2 alone compared to control cells. [file 10020_2022_470_MOESM3_ESM.pdf]

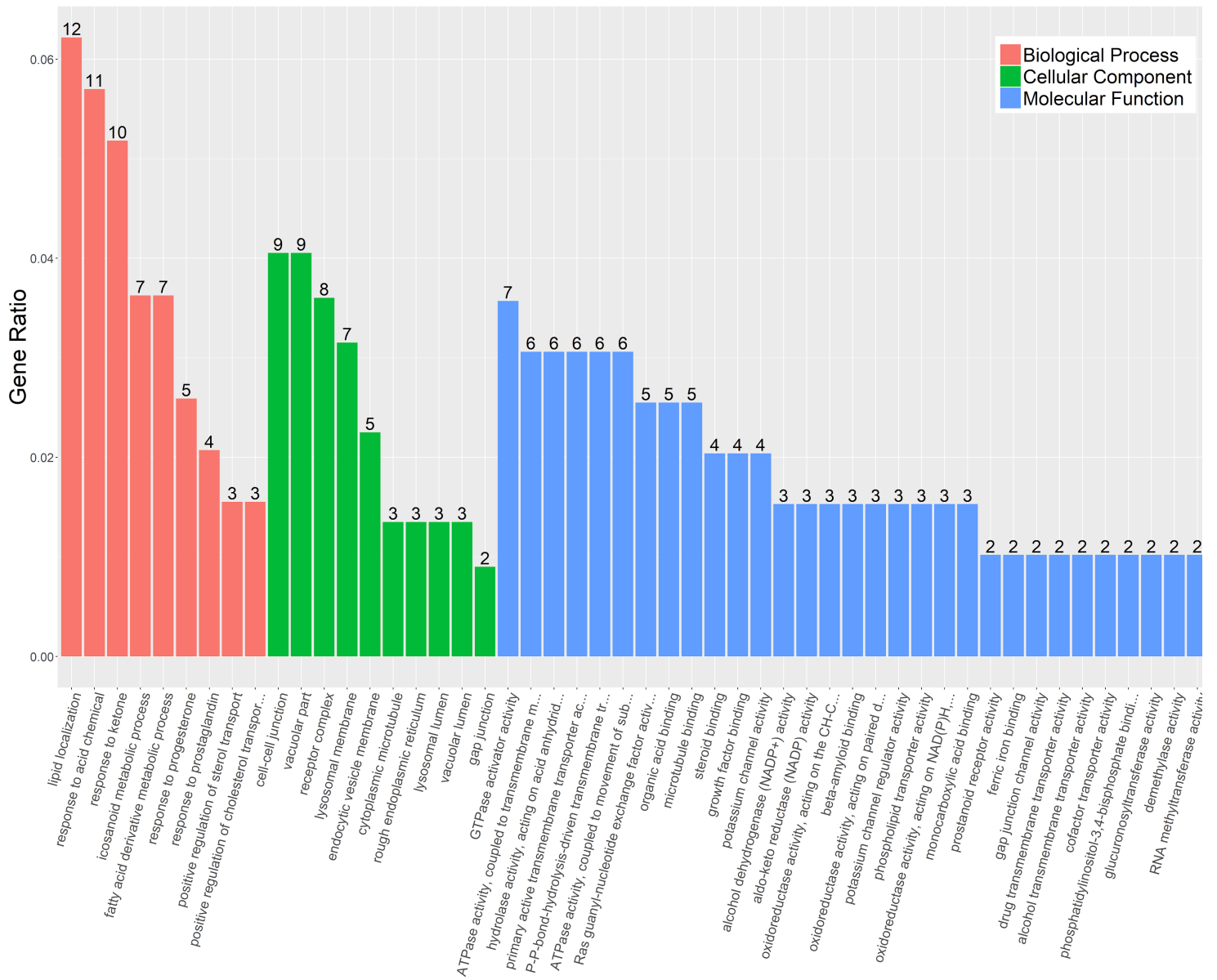

Supplement: Supplementary file 4 — Additional file 4: Figure S2. Gene Ontology (GO) analysis of the molecular function of genes. GO analysis shows that the molecular pathways regulated by the 2′,3′,4′-THC/E2 combination compared to E2 alone treated cells. The genes in green (cellular component) and blue (molecular function) bars were new, since they were regulated only by the combination. [file 10020_2022_470_MOESM4_ESM.pdf]
